# Critical Analysis of Metallothionein Immunohistochemistry for Diagnosis of Pediatric Wilson Disease: Histologic Scoring Facilitates Distinction from Clinical Mimics

**DOI:** 10.3390/diagnostics16182897

**Published:** 2026-09-09

**Authors:** Dua Abuquteish, Geraldine Garcia, Eve A. Roberts, Iram Siddiqui

**Affiliations:** 1 Department of Microbiology, Pathology and Forensic Medicine, Faculty of Medicine, The Hashemite University, Zarqa 13133, Jordan; dua@hu.edu.jo; 2 Division of Pathology, Department of Paediatric Laboratory Medicine, The Hospital for Sick Children, 555 University Ave, Black Wing Room 3207, Toronto, ON M5G 1X8, Canada; geraldine.garcia@sickkids.ca; 3 Departments of Paediatrics, Medicine, and Pharmacology & Toxicology, University of Toronto, Toronto, ON M5S 1A8, Canada; eve.roberts@dal.ca; 4 History of Science and Technology Programme, University of King’s College, Halifax, NS B3H 2A1, Canada

**Keywords:** metallothionein immunohistochemistry, Wilson disease, histologic scoring, copper toxicity, liver biopsy

## Abstract

**Background/Objectives:** Wilson disease (WD) is difficult to diagnose in children because it can mimic other pediatric liver diseases. It lacks specific routine histologic features. Recent studies suggest that metallothionein (MT) immunohistochemistry (IHC) may aid diagnosis, but pediatric data remain limited. **Methods:** This is a retrospective study of 121 pediatric liver biopsies, including WD (*n* = 63), primary sclerosing cholangitis (*n* = 23), metabolic dysfunction-associated steatotic liver disease (*n* = 19), autoimmune hepatitis (*n* = 13), and multidrug resistance protein 3 deficiency (*n* = 3). MT IHC was assessed for extent, intensity, pattern, and distribution. **Results:** MT positivity was identified in 96.8% of WD cases compared with 73.9% of PSC, 38.5% of AIH, and 10.5% of MASLD cases (*p* < 0.001). In WD, MT IHC characteristically demonstrated diffuse non-zonal cytoplasmic staining involving ≥25% of hepatocytes, most frequently >50%, with moderate-to-strong intensity. A threshold of ≥25% positive hepatocytes achieved the highest overall diagnostic accuracy (87.6%), with 80.9% sensitivity and 94.8% specificity. Diffuse non-zonal staining demonstrated 100% specificity in this cohort. **Conclusions:** MT IHC is a sensitive adjunctive marker for pediatric WD. Interpretation of staining extent, intensity, and distribution improves diagnostic specificity and assists in distinguishing WD from histologic mimics and cholestatic disorders.

## 1. Introduction

Wilson disease (WD) is a rare yet treatable disorder that alters copper metabolism in the liver, leading to toxic copper accumulation. Inheritance is autosomal recessive, with an estimated prevalence of 1:30,000 [1]. It is associated with variants in the *ATP7B* gene encoding the P-type ATPase, ATP7B [2]. WD may present at any age, with an average age at diagnosis of 13.2 years reported in a cohort of 133 patients, and it can present before the age of 5 years [3,4]. Establishing a diagnosis in this preschool age bracket can be difficult. In general, diagnosis is challenging because the clinical presentation is highly variable, encompassing liver disease, neurological and/or hematologic disorders, and psychiatric manifestations. Liver disease is often the first clinical manifestation and appears to be more prevalent in pediatric and young adult patients [2]. In contrast, adults typically present with neuropsychiatric manifestations and less prominent hepatic disease.

No single test diagnoses WD with certainty. The diagnostic assessment includes symptom-based scoring systems, biochemical tests of copper metabolism, and molecular analysis of *ATP7B* [5,6]. Scoring systems, such as the Leipzig score, are used and validated for both age groups [6]. However, if no definitive diagnosis is reached, a liver biopsy with copper quantification is required [5,6]. Hepatic copper content > 250 μg (4 μmol)/g dry weight is regarded as highly informative. However, this measurement requires adequate tissue obtained by liver biopsy, is unavailable at many centers, and often has a long turnaround time [7]. Liver biopsy may reveal histological findings supporting the diagnosis of WD or support an alternative diagnosis. It also provides the stage and grade of liver injury. Nevertheless, the challenge lies in the lack of histologic features specific to WD. Moreover, the clinical presentation can resemble that of other liver diseases, specifically autoimmune hepatitis (AIH) and metabolic dysfunction-associated steatotic liver disease (MASLD), previously referred to as non-alcoholic fatty liver disease (NAFLD). Histological findings on routine light microscopy may also resemble those disorders.

While ultrastructural examination by means of electron microscopy (EM) can yield highly specific indicators of mitochondrial abnormalities, thereby effectively differentiating WD from AIH and MASLD [8], certain limitations exist. EM requires fixing biopsy tissue in glutaraldehyde rather than the commonly used formalin, and it requires costly equipment and experienced technical skill sets for interpretation. Most importantly, the turnaround time for the diagnosis may take 7–10 days or more.

Timely and accurate diagnosis of WD is crucial, particularly in cases involving acute liver failure or end-stage liver disease, both of which may necessitate liver transplantation. Even without the urgency imposed by liver failure, early and accurate diagnosis of WD is important because starting effective treatment as early as possible has been shown to produce the best long-term outcomes.

Metallothioneins (MTs) are a family of proteins rich in cysteine. Within the cell, MTs bind essential heavy metals, such as copper and zinc, and play a crucial role in their homeostasis and detoxification. In children with WD at an early stage, copper within hepatocytes is bound to hepatocellular MT and thus cannot be detected by routine histological stains, despite increased copper concentration. Hepatic MT concentrations have been shown to be significantly elevated in patients with WD compared with controls [9,10] and in the untreated Jackson toxic milk mouse, a model for WD (Roberts, E.A. observations from unpublished data). Recent studies have used immunohistochemistry (IHC) to demonstrate hepatocellular MT expression, primarily in adult WD cohorts compared with adult controls [11,12,13,14]. These studies included only a limited number of pediatric patients. Therefore, a comprehensive evaluation of MT expression profiles in pediatric WD is needed. While data from adult studies are valuable, it is critical to avoid assuming that these findings directly translate to pediatric WD.

Herein, we aimed to examine MT IHC profiles in pediatric WD patients and compare them with those in children with relevant diseases in the differential diagnosis of WD: AIH, MASLD, primary sclerosing cholangitis (PSC), and Multidrug Resistance 3 deficiency (MDR3, also known as PFIC3). These diseases either present with overlapping histologic phenotypes, such as MASLD and AIH, or are known to cause copper accumulation in hepatocytes due to inadequate bile flow (chronic cholestasis). In addition, we aimed to expand the assessment of MT staining patterns in pediatric patients. Looking beyond parameters previously reported (percentage and intensity), we examined patterns (granular and cytoplasmic) and distribution within liver zones. Finally, we evaluated MT IHC expression in treated and untreated WD patients.

## 2. Materials and Methods

This is a retrospective cohort study evaluating and analyzing the utility of MT IHC staining in diagnosing WD on liver biopsies in children who presented between 1980 and 2023 (age < 18 years at the time of diagnosis). The Research Ethics Board approved this study at the Hospital for Sick Children (REB# 1000055763—study received original REB approval on 25 January 2018). The criteria for including these patients were hepatic tissue copper quantification > 250 µg/g dry weight or genetic confirmation of WD.

MT IHC was performed on all Formalin-Fixed Paraffin-Embedded (FFPE) tissue liver specimens. Cases included WD and disease controls (AIH, PSC, MASLD, and MDR3 deficiency), where clinical diagnoses were established according to accepted clinicopathologic criteria and clinical data. MT IHC scoring was performed independently by two gastrointestinal and hepatopathologists (I.S. and D.A.) who were blinded to the clinical diagnosis. In addition to MT IHC, trichrome stain to stage fibrosis and orcein stain for copper-associated protein (CAP) were performed and scored. Cases with insufficient tissue for IHC were excluded.

MT IHC staining was scored as follows: no staining (score 0), <25% staining (score 1), 25–50% staining (score 2), and >50% staining (score 3). The intensity of MT staining was scored as mild, moderate, or strong. MT staining patterns were categorized into three categories: cytoplasmic, granular, and cytoplasmic with granular. MT staining distribution was described as pericentral, periportal, non-zonal (patchy), and non-zonal (diffuse). Orcein stain for CAP was scored as no staining (score 0), periportal staining (score 1), and non-zonal staining (score 2). Trichrome stain was used to assess fibrosis, which was staged according to the Batts–Ludwig fibrosis staging system (F0–F4), categorized as no fibrosis (score 0), mild-stage fibrosis (scores F1 and F2), and advanced fibrosis (scores F3 and F4).

Staining and immunohistochemistry (IHC): H&E (hematoxylin and eosin), orcein, and Masson’s trichrome staining were performed following the standard histopathological techniques. Metallothionein IHC staining was performed using a rabbit polyclonal anti-Metallothionein antibody (Abcam, Cambridge, UK, Cat. No. ab12228) at a dilution of 1:200. Staining was carried out on the Dako Omnis platform using Dako reagents (Dako, Glostrup, Denmark). Heat-induced epitope retrieval was performed using high-pH buffer for 30 min. This was followed by a 30 min protein blocking step, 30 min primary antibody incubation, and 30 min detection using the Omnis detection system.

Statistical analysis: Categorical variables are presented as frequencies and percentages. Interobserver agreement for MT IHC scoring was assessed using weighted Cohen’s κ statistics with 95% confidence intervals and interpreted according to the Landis and Koch classification. Differences in MT IHC staining characteristics among the diagnostic groups (WD, PSC, MASLD, AIH, and MDR3 deficiency) were evaluated by using Fisher’s exact test. Secondary analyses were performed to assess the associations between fibrosis stage and MT staining characteristics, fibrosis stage and Orcein staining pattern, and Orcein staining pattern and MT staining distribution. For contingency tables larger than 2 × 2 or when exact computation was computationally intensive, two-sided Fisher’s exact test with Monte Carlo simulation (100,000 replicates) using the statistical package was used to estimate exact *p* values. Weighted Cohen’s κ was calculated using the DescTools package. *p* values for related multiple comparisons were adjusted using the Benjamini–Hochberg (BH) false discovery rate method. A two-sided *p* value < 0.05 was considered statistically significant. All statistical analyses were performed using R version 4.6.1 (R Foundation for Statistical Computing, Vienna, Austria).

## 3. Results

### 3.1. Study Population

The baseline characteristics of the study cohort, including age and sex distributions within each diagnostic group, are summarized in Table 1. A total of 121 liver specimens were included, comprising 109 (90.1%) needle biopsies, 11 (9.1%) explants, and 1 (0.8%) wedge biopsy. The cohort consisted of 63 patients with WD and 58 disease controls, including 23 PSC, 19 MASLD, 13 AIH, and 3 MDR3 deficiency cases. Of the 63 WD cases, 53 (84.1%) were treatment-naïve and 10 (15.9%) were obtained after treatment. Among the treated cases, treatment duration was less than 1 month in 2 (20.0%) cases, between 1 month and less than 1 year in 1 (10.0%) case, and more than 1 year in 7 (70.0%) cases.

Fibrosis stage was assessed for all cases and was classified as no fibrosis in 14 (11.6%), low-stage fibrosis in 39 (32.2%), and advanced fibrosis in 68 (56.2%). Orcein stain was reviewed for 78 evaluable cases, of which 34 (43.6%) showed negative staining, 34 (43.6%) demonstrated a periportal staining pattern, and 10 (12.8%) exhibited a non-zonal staining pattern.

### 3.2. Interobserver Agreement

MT IHC staining was evaluated independently by two pathologists (D.A. and I.S.) for interobserver agreement, including stain percentage, intensity, pattern, and distribution. The fibrosis stage and orcein stain were evaluated by a single pathologist (D.A.). Interobserver agreement between the two pathologists (D.A. and I.S.) was assessed for each ordinal MT IHC parameter using quadratically weighted Cohen’s kappa (κ) with Fleiss–Cohen weights and 95% confidence intervals (CIs). Observed agreement was also calculated.

Interobserver agreement for MT IHC staining is summarized in Table 2. Weighted Cohen’s κ values demonstrated almost perfect agreement for the assessment of MT-positive tissue (κ = 0.928, 95% CI: 0.877–0.979), staining intensity (κ = 0.927, 95% CI: 0.893–0.961), and staining distribution (κ = 0.864, 95% CI: 0.785–0.943), with corresponding observed agreement rates of 84.3%, 81.0%, and 78.5%, respectively. In contrast, agreement for staining pattern was moderate (κ = 0.587, 95% CI: 0.454–0.721), with an observed agreement of 75.2%.

Cases with discrepant MT scores were subsequently reviewed jointly and a consensus was reached. The consensus scores were used for all subsequent statistical analyses.

### 3.3. MT Positivity Across WD and Controls

The frequency of MT IHC positivity among patients with WD and the control groups is shown in Table 3. MT IHC positivity differed significantly between the diagnostic groups (Fisher’s exact test, *p* < 0.001). MT positivity was highest in WD (96.8%) and MDR3 deficiency (100.0%), whereas lower positivity rates were observed in PSC (73.9%), AIH (38.5%), and MASLD (10.5%).

### 3.4. Percentage of MT-Positive Hepatocytes Across WD and Controls

The percentage of MT-positive hepatocytes across WD and control groups is summarized in Table 4. The proportion of MT-positive tissue differed significantly among the diagnostic groups (*p* < 0.001). More than 50% of MT-positive hepatocytes were observed predominantly in WD cases (46/63, 73.0%), whereas most PSC cases exhibited <25% MT-positive tissue (17/23, 73.9%). In contrast, the majority of MASLD (17/19, 89.5%) and AIH (8/13, 61.5%) cases showed no MT staining. All three MDR3 deficiency cases demonstrated MT positivity, with one case showing <25% positive tissue and two cases showing 25–50% positivity.

### 3.5. MT Staining Intensity Across WD and the Control Groups

**Figure 1:**
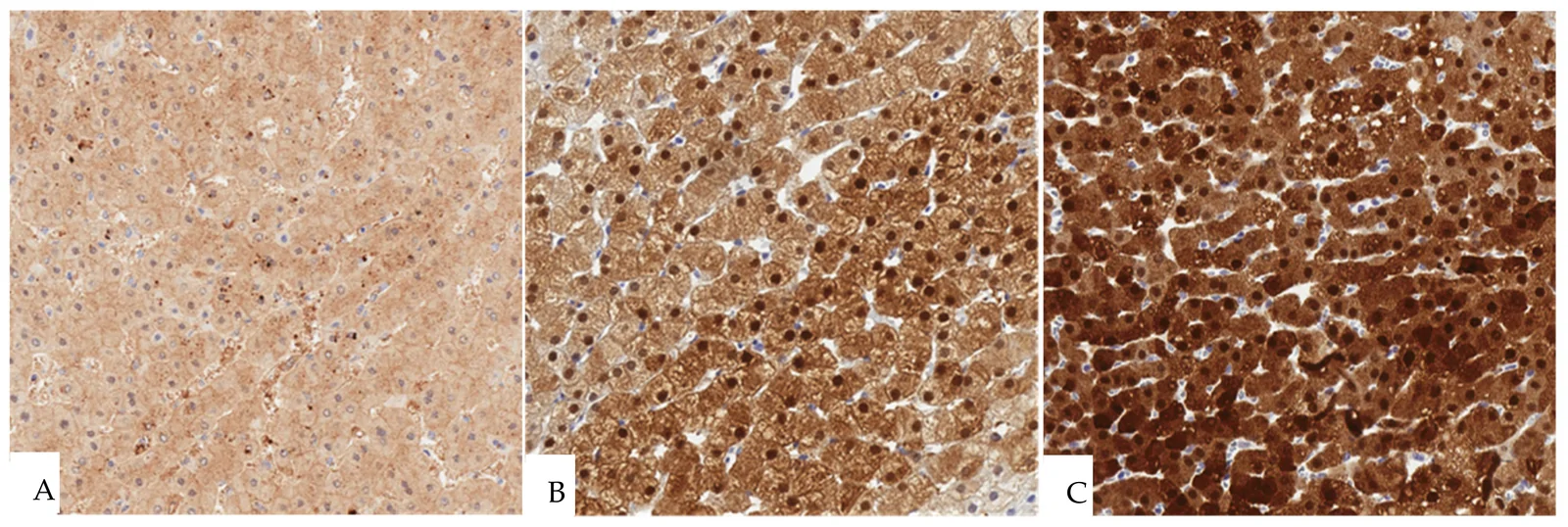
Representative MT IHC staining intensity: (**A**) Mild cytoplasmic MT staining, (**B**) moderate cytoplasmic MT staining, and (**C**) strong cytoplasmic MT staining in hepatocytes. Whole-slide images scanned at ×40.

The intensity of MT IHC staining across WD and the control groups is shown in Table 5, with representative photomicrographs illustrating the staining intensity categories presented in Figure 1. Staining intensity differed significantly among the diagnostic groups (*p* < 0.001). Strong MT staining was the predominant pattern in WD (39/63, 61.9%) and was observed in all three of the three MDR3 deficiency cases. In contrast, most MASLD (17/19, 89.5%) and AIH (8/13, 61.5%) cases showed no MT staining, whereas PSC cases most commonly demonstrated mild staining (11/23, 47.8%).

### 3.6. MT Staining Pattern Across WD and Control Groups

**Figure 2:**
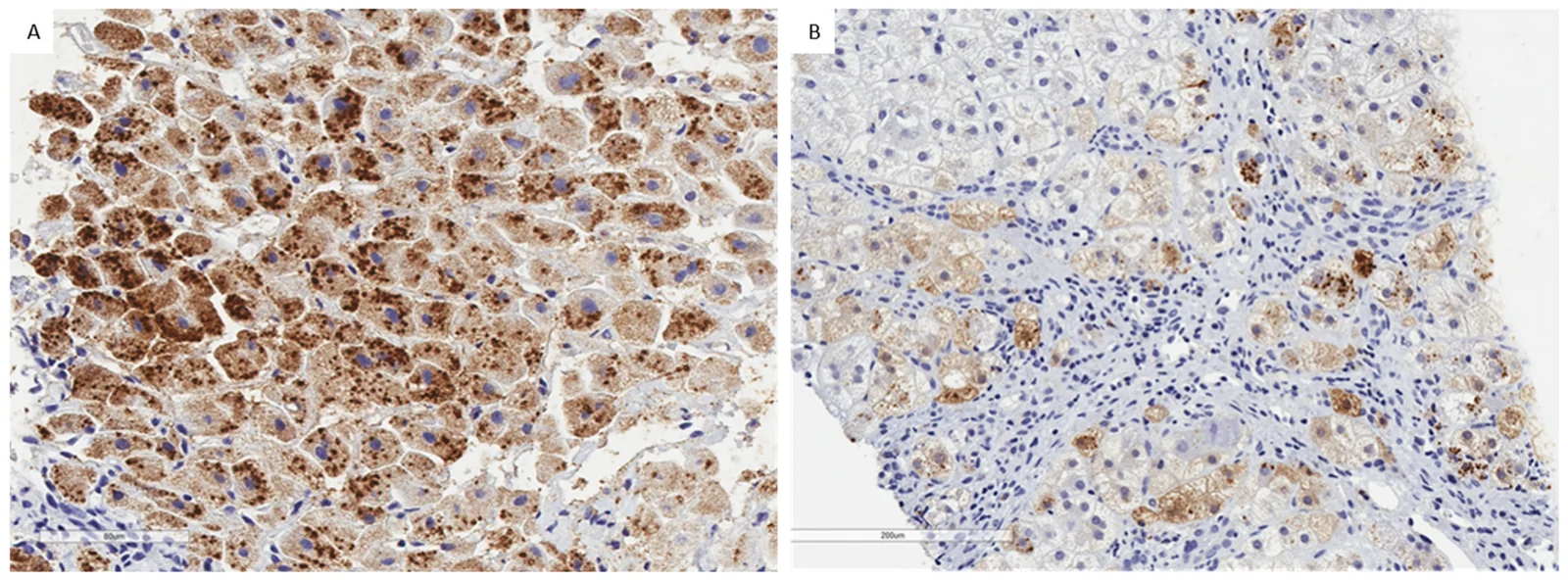
Representative MT IHC staining patterns. (**A**) WD. (**B**) MDR3 deficiency. Both cases demonstrate combined cytoplasmic and granular staining. Whole-slide images scanned at ×40.

The MT IHC staining pattern across WD and the control groups is shown in Table 6, with representative examples of the combined cytoplasmic and granular staining pattern illustrated in Figure 2. The MT IHC staining pattern differed significantly among the diagnostic groups (*p* < 0.001). Cytoplasmic staining was the predominant pattern in WD (56/63, 88.9%), whereas cytoplasmic and granular staining predominated in PSC (10/23, 43.5%) and was present in all three of the three MDR3 cases. Granular staining alone was uncommon across all diagnostic groups.

### 3.7. MT Staining Distribution Across WD and Control Groups

**Figure 3:**
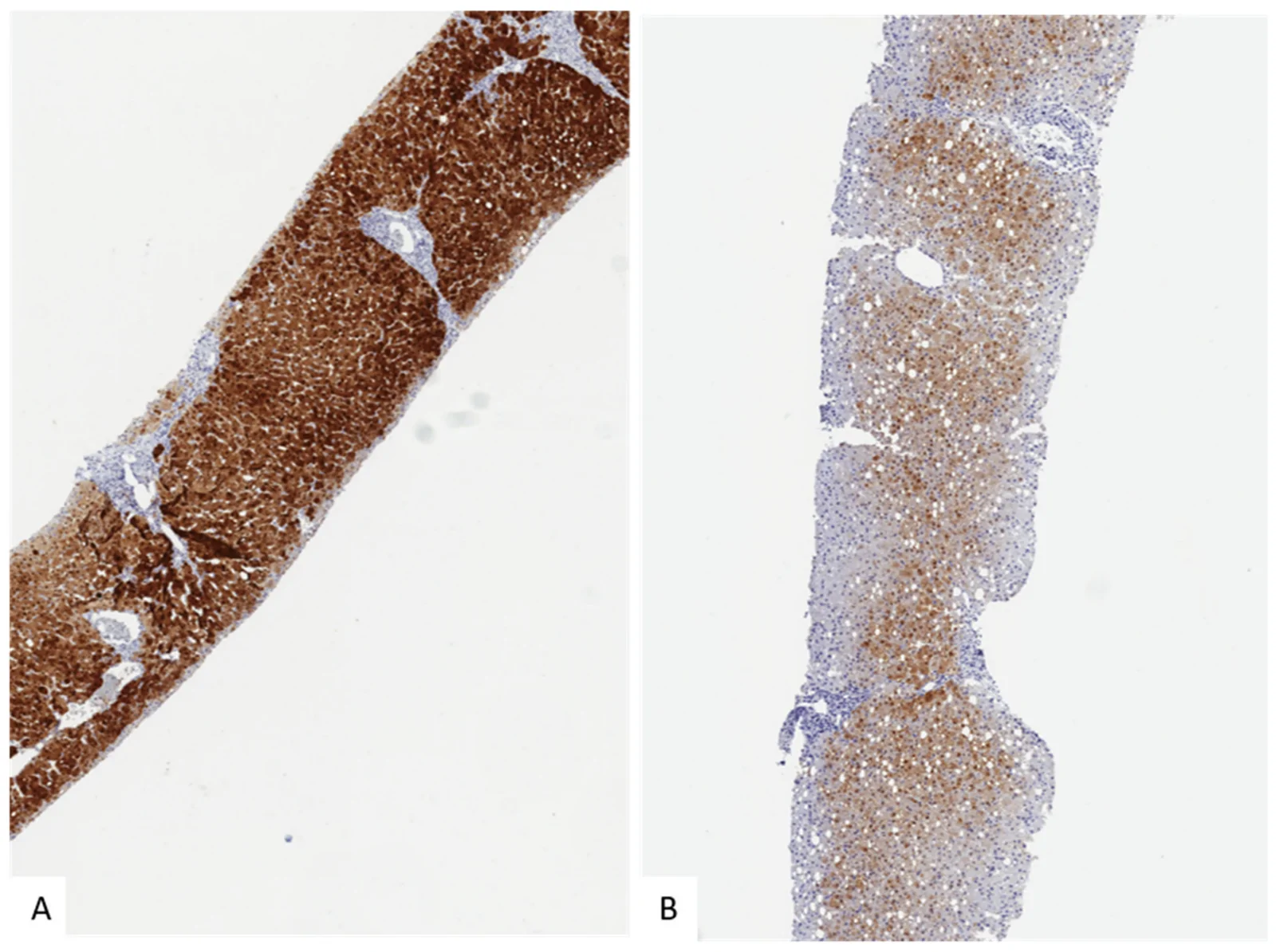
Representative MT IHC staining distribution in WD. (**A**) Diffuse non-zonal staining. (**B**) Patchy non-zonal staining. Whole-slide images scanned at ×20.

**Figure 4:**
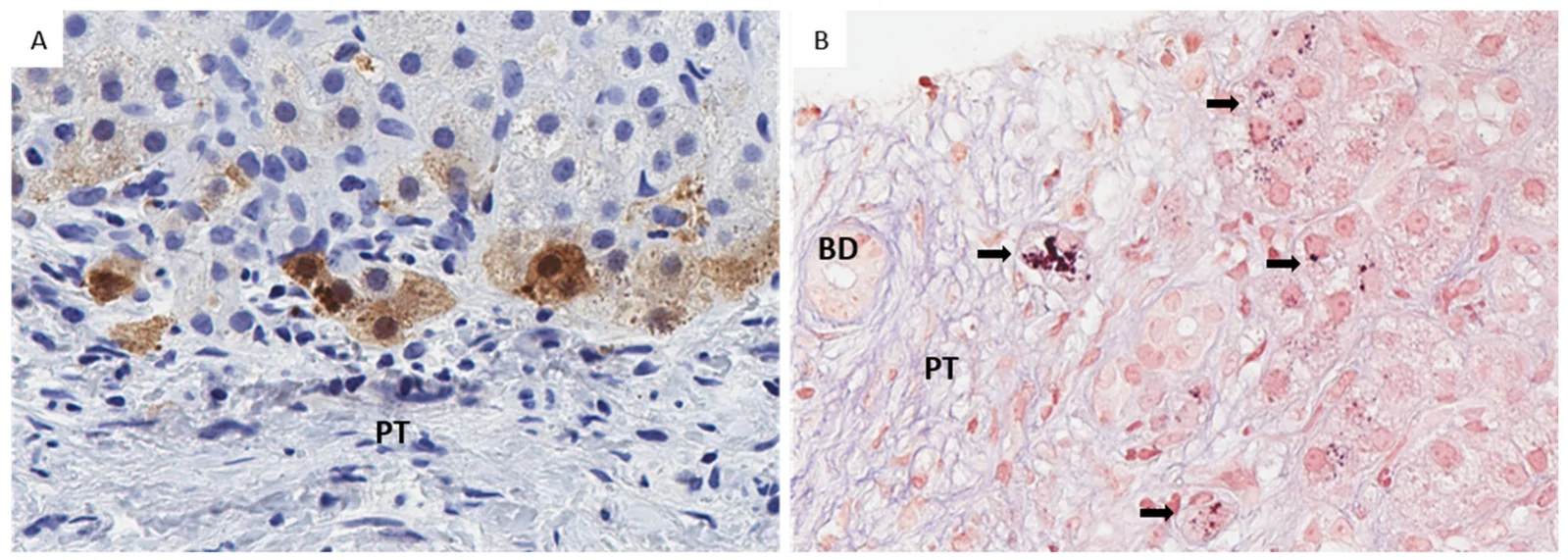
Representative PSC case. (**A**) MT IHC showing periportal staining with combined cytoplasmic and granular staining. (**B**) Corresponding orcein stain from the same area showing positive copper-associated protein (CAP) deposition (black arrows). Whole-slide images scanned at ×40. PT: portal tract. BD: bile duct.

The MT IHC staining distribution within the different zones across WD and control groups is summarized in Table 7. The zonal distribution of MT staining differed significantly among the diagnostic groups (*p* < 0.001). Diffuse non-zonal staining was the predominant pattern in WD (35/63, 55.6%), whereas periportal staining was most frequently observed in PSC (12/23, 52.2%) and MDR3 deficiency (2/3, 66.7%). In contrast, most MASLD and AIH cases showed absent MT staining, while the remaining positive cases exhibited a patchy non-zonal distribution. Representative examples of diffuse non-zonal and patchy non-zonal staining in WD are shown in Figure 3 and periportal MT staining with corresponding orcein staining in PSC is shown in Figure 4.

### 3.8. Diagnostic Performance of MT Immunohistochemistry for WD

The overall diagnostic performance of MT IHC staining for the diagnosis of WD is presented in Table 8. The diagnostic performance of MT IHC varied according to the definition of a positive test. Using any MT positivity as the diagnostic criterion yielded the highest sensitivity (96.8%) but only moderate specificity (53.4%). Increasing the threshold to ≥25% MT-positive hepatocytes substantially improved specificity to 94.8% while maintaining a sensitivity of 80.9%, resulting in the highest overall diagnostic accuracy (87.6%). A threshold of >50% further increased specificity to 98.3% and the positive predictive value to 97.9%, although sensitivity decreased to 73.0%. Strong staining, diffuse non-zonal staining, and the combination of strong and diffuse staining progressively increased specificity at the expense of sensitivity, with diffuse non-zonal staining and strong plus diffuse staining achieving 100% specificity and positive predictive value within the present cohort. A threshold of ≥25% MT-positive hepatocytes provided the highest overall diagnostic accuracy, while diffuse non-zonal staining demonstrated the greatest specificity. The combination of ≥25% hepatocyte staining and diffuse non-zonal staining yielded a sensitivity of 55.6% and specificity of 100%, with an overall accuracy of 76.9%.

### 3.9. Association Between Fibrosis Stage and MT IHC Characteristics in WD

As shown in Table 9, MT immunoreactivity was nearly ubiquitous across the WD cohort and was present in all cases without fibrosis. Greater MT expression, defined as staining in ≥25% or >50% of hepatocytes, was observed predominantly in cases with low-stage and advanced fibrosis. These associations remained statistically significant after Benjamini–Hochberg correction (adjusted *p* = 0.042 for both comparisons). In contrast, after multiple-testing correction, no significant association was observed between staining pattern (non-zonal patchy versus non-zonal diffuse) and advanced fibrosis. These findings suggest that the extent of MT expression, rather than its mere presence or distribution pattern, is associated with hepatic fibrosis and may reflect early fibrogenic injury. Among the 23 PSC cases, MT positivity was observed in 17 (73.9%). Fibrosis severity among MT-positive cases ranged from no fibrosis (1/17) to low-stage fibrosis (7/17) and advanced fibrosis (9/17), indicating that MT expression occurred across the full spectrum of fibrosis stages in PSC.

Most of the AIH cases had some degree of fibrosis; of them, 1/13 (7.7%) had no fibrosis, 4/13 (30.8%) had low-stage fibrosis, and 8/13 (61.5%) had advanced fibrosis. There was no extensive MT staining in any of the AIH cases. Only 5 of the 13 AIH cases showed ≥25% MT staining and 4 of 5 had advanced fibrosis while 1 of 5 had stage 2 fibrosis.

MT immunoreactivity was uncommon in MASLD, with only 2/19 cases showing positive staining. One case demonstrated ≥25% staining and one >50% staining; these cases had no fibrosis and low-stage fibrosis, respectively. Overall, 6/19 (31.6%) MASLD cases had no fibrosis, 10/19 (52.6%) had low-stage fibrosis, and 3/19 (15.8%) had advanced fibrosis. All three MDR3 deficiency cases exhibited advanced fibrosis.

### 3.10. Association Between Fibrosis Stage and Orcein Staining Pattern

Orcein stain was reviewed for 78 of the 121 study cases (64.5%). In the remaining 43 cases (35.5%), the original stain was faded and not interpretable and a repeat stain could not be performed due to limited tissue in the block. Among the evaluable cases, 35 (44.3%) demonstrated no orcein staining, 34 (43.0%) exhibited a periportal staining pattern, and 10 (12.7%) showed a non-zonal staining pattern. The evaluable cohort consisted of 54 WD cases, 23 PSC cases, and 1 MDR3 deficiency case.

The association between fibrosis stage and the pattern of orcein staining is summarized in Table 10. Orcein staining pattern differed significantly according to fibrosis stage (*p* = 0.009). Negative staining was the most common pattern in both the no fibrosis (66.7%) and low-stage fibrosis (64.0%) groups, whereas periportal staining became increasingly frequent with advancing fibrosis, accounting for 33.3%, 36.0%, and 48.9% of cases in the no fibrosis, low-stage fibrosis, and advanced fibrosis groups, respectively. Notably, non-zonal staining was observed exclusively in patients with advanced fibrosis, where it was present in 21.3% of cases. However, this finding should be interpreted in the context of the mixed study cohort, which included both WD and non-WD cases. Overall, the proportion of cases with negative orcein staining decreased as fibrosis severity increased, while periportal and non-zonal staining patterns became more prevalent, suggesting greater accumulation of copper-associated protein with progressive hepatic fibrosis.

### 3.11. Association Between Orcein Staining Pattern and MT Staining Distribution

The association between orcein staining pattern and MT staining distribution is summarized in Table 11. MT staining distribution differed significantly according to the orcein staining pattern (*p* = 0.002). Cases with negative orcein staining most commonly demonstrated non-zonal diffuse MT staining (58.8%), whereas periportal orcein staining was associated with a broader distribution of MT staining, including periportal (35.3%) and non-zonal patchy (32.4%) patterns. In contrast, non-zonal orcein staining was predominantly associated with non-zonal patchy (50.0%) and non-zonal diffuse (40.0%) MT staining, with no cases demonstrating absent MT staining.

### 3.12. Effect of Treatment on MT Staining in WD

The effect of therapy on MT IHC staining is summarized in Table 12. Of the 63 WD cases, 53 (84.1%) were treatment-naïve and 10 (15.9%) were obtained after zinc therapy. MT staining intensity differed between treatment-naïve and post-treatment cases on unadjusted analysis (*p* = 0.013); however, this difference did not remain statistically significant after BH correction (adjusted *p* = 0.065). Strong MT staining was observed in 67.9% of treatment-naïve cases compared with 30.0% of post-treatment cases, while mild staining increased from 7.5% to 40.0% following treatment. Moderate staining was observed at similar frequencies in both groups (22.6% vs. 20.0%). Although not statistically significant, treatment was associated with a trend toward a lower proportion of MT-positive hepatocytes, with >50% positive hepatocytes observed in 77.4% of treatment-naïve cases compared with 50.0% of post-treatment cases (*p* = 0.056). No significant differences were identified in overall MT positivity (*p* = 0.294), MT staining pattern (*p* = 0.483), or MT staining distribution (*p* = 0.509). These findings suggest that therapy is associated with reduced MT staining intensity, while overall MT expression and staining characteristics remain largely preserved.

## 4. Discussion

Metallothioneins, first identified in 1957, are cysteine-rich proteins involved in intracellular copper sequestration and detoxification [14,15]. In WD, impaired ATP7B-mediated copper excretion results in hepatocellular copper accumulation and subsequent MT upregulation, providing the biological rationale for MT immunohistochemistry as a marker of hepatic copper overload. Previous studies have demonstrated high sensitivity of MT IHC for WD [11,12,13], but pediatric data remain limited. In this study, one of the largest pediatric series to date, we evaluated MT expression in WD and compared it with both histologic mimics and cholestatic disorders associated with secondary copper accumulation.

Our results are consistent with previous studies by Stokes et al., Rowan et al., and Wiethoff et al. [11,12,13] by demonstrating the diagnostic value of staining extent, intensity, and distribution in a pediatric population. However, our study expands upon these observations in several important ways. First, we focused exclusively on a pediatric population. Second, we evaluated the disease controls that were either clinical mimics of pediatric WD such as MASLD or conditions that may exhibit secondary copper accumulation, including PSC and MDR3 deficiency. Third, we systematically assessed staining pattern and zonal distribution in addition to staining extent and intensity. These additional parameters provided valuable discriminatory information and helped distinguish primary copper overload due to WD from secondary copper retention associated with cholestatic disorders. In our cohort, granular staining was uncommon in WD but frequently observed in PSC, suggesting that this feature may be a useful clue favoring secondary copper retention.

The findings from our study confirm the high sensitivity of MT IHC for pediatric WD and demonstrate that diagnostic value lies not merely in MT positivity but in the overall staining profile. While MT expression was identified in most WD cases, it was also observed in cholestatic disorders. However, WD showed a characteristic pattern of extensive moderate-to-strong cytoplasmic staining with diffuse non-zonal distribution, whereas PSC and MDR3 deficiency generally demonstrated more limited staining, periportal predominance, and frequent combined granular staining. A threshold of ≥25% MT-positive hepatocytes provided the best balance of sensitivity and specificity. These findings suggest that interpretation of MT IHC should move beyond a binary positive-versus-negative approach and incorporate quantitative and distributional features of staining.

Our findings differ somewhat from those reported by Wiethoff et al. [13], who observed a prominent perivenular MT staining pattern in multiple liver diseases with the exception of alcohol-associated steatotic liver disease and MASLD. Instead, pediatric cases demonstrated patchy or diffuse non-zonal MT staining, suggesting potential differences between pediatric and adult liver disease. Treatment was associated with reduced staining intensity but largely preserved staining pattern and distribution, although the limited number of treated cases precludes definitive conclusions. Whether these differences reflect age-related biology, disease stage, or technical factors remains uncertain and warrants further investigation. Importantly, liver biopsies of two children with WD in our cohort showed no MT expression by immunohistochemistry, highlighting that negative MT IHC results require contextualization with other clinical and laboratory findings.

Distinguishing primary copper overload in WD from secondary copper retention in cholestatic liver disease can be challenging. In our cohort, MT positivity was common in PSC and MDR3 deficiency; however, the staining characteristics differed substantially from those seen in WD. Particularly notable was the frequent granular staining pattern observed in PSC, which was uncommon in WD. Although the biological basis of this finding remains uncertain, it may reflect differences in intracellular copper handling and suggests that prominent granular staining should prompt consideration of cholestatic liver disease rather than WD [11,12,13].

Increasing fibrosis stage was associated with stronger and more extensive MT expression in WD, likely reflecting cumulative hepatic copper exposure and chronic oxidative stress. Similarly, diffuse non-zonal deposition of copper-associated proteins identified by means of orcein staining was restricted to advanced fibrosis in general. These findings support the concept that hepatic copper deposition evolves during disease progression and are consistent with the observation that excess copper is initially sequestered by hepatocellular MT before accumulating as lysosomal copper-associated proteins detectable by conventional histochemical stains [16]. The presence of diffuse MT staining in cases with absent or limited orcein positivity further highlights the complementary value of MT IHC in detecting hepatic copper overload. This relationship with fibrosis contrasts with the study by Stokes et al. in which the authors reported that cases with advanced fibrosis had a sensitivity of 88.2%, while those without advanced fibrosis had a sensitivity of 100% [11]. The authors in this study specifically included chronic cholestatic diseases as controls, predominantly PSC (34 cases) and other chronic biliary tract diseases (8 cases). They found that all 42 cholestatic control cases, including all PSC cases, were negative for MT IHC, using their predefined positive threshold. Our study in a pediatric-specific cohort identifies low-level, predominantly periportal/granular MT staining in many PSC cases, most with low- or advanced-stage fibrosis. This underscores the need to apply caution and analyze MT IHC staining characteristics in greater detail and not as a binary threshold.

This study is limited by its retrospective design and the small number of certain comparator groups, particularly MDR3 deficiency. This limits conclusions regarding the full spectrum of MT expression in this disorder. Several rare pediatric copper-related disorders, such as Indian childhood cirrhosis, were not represented. Third, the retrospective design may introduce selection bias.

Despite the limitations, the study has several notable strengths, including a relatively large pediatric WD cohort, excellent interobserver reproducibility, and the inclusion of clinically relevant disease controls. Collectively, the findings validate MT IHC as a robust adjunctive diagnostic tool in pediatric WD and provide practical guidance for interpretation based on staining extent, intensity, and distribution. Importantly, although MT IHC demonstrated excellent diagnostic performance, it is not entirely disease-specific. A small number of non-WD cases exhibited substantial MT expression, further emphasizing that MT IHC should not be interpreted in isolation. Clinical, biochemical, genetic, and histologic findings remain essential for establishing the diagnosis of WD.

Interestingly, our findings parallel observations reported decades earlier (in 1989) by Elmes et al., who demonstrated abnormal MT expression in both WD and cholestatic disorders and noted characteristic differences in staining patterns among copper-retaining diseases [17]. The present study extends these observations through modern immunohistochemical methods and systematic evaluation of staining extent, intensity, and zonal distribution in a large pediatric cohort.

Overall, the strongest message of this study is not merely that MT is positive in WD, but that a distinct pediatric WD staining signature emerges when extent, intensity, and distribution are interpreted together. Among the evaluated diagnostic criteria, ≥25% hepatocyte staining provided the best balance of sensitivity and specificity, while diffuse non-zonal cytoplasmic expression showed the highest specificity.

## 5. Conclusions

Metallothionein immunohistochemistry is an efficient and valuable adjunctive tool for the diagnosis of pediatric WD, which can be performed on an initial liver biopsy. Diagnostic accuracy is optimized by evaluating staining extent, intensity, and distribution rather than MT positivity alone. We have shown that while ≥25% hepatocyte MT staining provides the best overall diagnostic accuracy, a diffuse non-zonal cytoplasmic staining pattern adds a highly specific feature for the diagnosis of WD. Moreover, although not entirely disease-specific, MT IHC can improve diagnostic confidence and assist in distinguishing WD from cholestatic disorders and other histologic mimics when interpreted in conjunction with clinical, biochemical, genetic, and histologic findings.

## Figures and Tables

**Table 1:** Baseline characteristics of the study population.

Characteristic	*n* (%)	Age, Mean ± SD, Years	Male, *n* (%)	Female, *n* (%)
Total number of cases	121	-	65 (53.7%)	56 (46.3%)
Diagnosis, *n* (%)				
WD	63 (52.1%)	11.3 ± 3.9	32 (50.8%)	31 (49.2%)
PSC	23 (19.0%)	13.2 ± 4.4	16 (69.6%)	7 (30.4%)
MASLD	19 (15.7%)	11.2 ± 2.0	13 (68.4%)	6 (31.6%)
AIH	13 (10.7%)	8.8 ± 4.7	4 (30.8%)	9 (69.2%)
MDR3 deficiency	3 (2.5%)	7.7 ± 1.5	0/3	3/3
WD-treatment status				
Treatment-naïve	53 (84.1)			
Post-treatment	10 (15.9)			

**Table 2:** Interobserver agreement for MT IHC parameters using weighted Cohen’s κ statistics.

Parameter	Weighted Cohen’s κ	95% CI	Observed Agreement	Interpretation
MT positive tissue	0.928	0.877–0.979	84.3%	Almost perfect
Intensity of staining	0.927	0.893–0.961	81%	Almost perfect
Pattern of staining	0.587	0.454–0.721	75.2%	Moderate
Staining distribution	0.864	0.785–0.943	78.5%	Almost perfect

**Table 3:** Frequency of MT-positive cases among patients with WD and the control groups.

Diagnosis	Patients, *n* (%)	MT-Positive Cases, *n* (%)
WD	63 (52.1%)	61 (96.8%)
MASLD	19 (15.7%)	2 (10.5%)
AIH	13 (10.7%)	5 (38.5%)
PSC	23 (19.0%)	17 (73.9%)
MDR3	3 (2.5%)	3 (100.0%)
Overall *p* value < 0.001; BH-adjusted *p* value < 0.001		

**Table 4:** Percentage of MT-positive tissue among patients with WD and the control groups.

Diagnosis	No Staining	<25% Staining	25–50% Staining	>50% Staining
WD (*n* = 63)	2	10	5	46
MASLD (*n* = 19)	17	1	0	1
AIH (*n* = 13)	8	5	0	0
PSC (*n* = 23)	6	17	0	0
MDR3 (*n* = 3)	0	1	2	0
Overall *p* value < 0.001; BH-adjusted *p* value < 0.001				

**Table 5:** Intensity of MT IHC staining among WD and control groups.

Diagnosis	No Staining	Mild Staining	Moderate Staining	Strong Staining
WD (*n* = 63)	2	8	14	39
MASLD (*n* = 19)	17	1	1	0
AIH (*n* = 13)	8	5	0	0
PSC (*n* = 23)	6	11	5	1
MDR3 (*n* = 3)	0	0	0	3
Overall *p* value < 0.001; BH-adjusted *p* value < 0.001				

**Table 6:** Pattern of MT IHC staining among WD and control groups.

Diagnosis	No Staining	Cytoplasmic	Granular	Cytoplasmic and Granular
WD (*n* = 63)	2	56	1	4
MASLD (*n* = 19)	17	1	0	1
AIH (*n* = 13)	8	5	0	0
PSC (*n* = 23)	6	5	2	10
MDR3 (*n* = 3)	0	0	0	3
Overall *p* value < 0.001; BH-adjusted *p* value < 0.001				

**Table 7:** MT IHC staining distribution among WD and control groups.

Diagnosis	No Staining	Pericentral	Periportal	Patchy (Non-Zonal)	Diffuse (Non-Zonal)
WD (*n* = 63)	2	0	1	25	35
MASLD (*n* = 19)	17	0	0	2	0
AIH (*n* = 13)	8	0	0	5	0
PSC (*n* = 23)	6	0	12	5	0
MDR3 (*n* = 3)	0	0	2	1	0
Overall *p* value: <0.001; BH-adjusted *p* value < 0.001					

**Table 8:** Diagnostic performance of different MT IHC staining criteria for the diagnosis of WD.

Diagnostic Criterion	Definition	Sensitivity (%)	Specificity (%)	PPV (%)	NPV (%)	Accuracy (%)
AnyMT positivity	>0% staining	96.8	53.4	69.3	93.9	76.0
≥25% staining	≥25% of hepatocytes stained	80.9	94.8	94.4	82.1	87.6
>50% staining	>50% of hepatocytes stained	73.0	98.3	97.9	77.0	85.1
Strong staining	Strong intensity	61.9	93.1	90.7	69.2	76.9
Diffuse non-zonal staining	Diffuse distribution	55.6	100.0	100.0	67.4	76.9
Strong +diffuse staining	Strong intensity and diffuse distribution	46.0	100.0	100.0	63.0	71.9
≥25% staining + diffuse non-zonal staining	≥25% of hepatocytes stained and diffuse staining	55.6	100.0	100.0	67.4%	76.9%

**Table 9:** Association between fibrosis stage and MT IHC staining in WD.

MT IHC Characteristic	No Fibrosis (*n* = 6)	Low-Stage Fibrosis (*n* = 15)	Advanced Fibrosis (*n* = 42)	*p* Value	BH-Adjusted *p* Value
Any MT staining	6 (100.0%)	14 (93.3%)	41 (97.6%)	0.558	0.558
MT staining ≥ 25%	2 (33.3%)	14 (93.3%)	35 (83.3%)	0.012	0.042
MT staining > 50%	2 (33.3%)	14 (93.3%)	30 (71.4%)	0.017	0.042
Non-zonal patchy staining	3 (50.0%)	2 (13.3%)	20 (47.6%)	0.049	0.082
Non-zonal diffuse staining	2 (33.3%)	12 (80.0%)	21 (50.0%)	0.078	0.098

**Table 10:** Association between fibrosis stage and orcein staining pattern.

Orcein Staining Pattern	No Fibrosis (*n* = 6)	Low-Stage Fibrosis (*n* = 25)	Advanced Fibrosis (*n* = 47)
Negative	4 (66.7%)	16 (64.0%)	14 (29.8%)
Periportal	2 (33.3%)	9 (36.0%)	23 (48.9%)
Non-zonal	0 (0.0%)	0 (0.0%)	10 (21.3%)
*p* value 0.009			

**Table 11:** Association between orcein staining pattern and MT staining distribution.

Orcein Pattern	No Staining	Periportal	Patchy	Diffuse
Negative	8.8%	2.9%	29.4%	58.8%
Periportal	11.8%	35.3%	32.4%	20.6%
Non-zonal	0.0%	10.0%	50.0%	40.0%

**Table 12:** Effect of treatment on MT IHC staining in WD.

Variable	Treatment-Naïve (*n* = 53)	Post-Treatment (*n* = 10)	*p* Value	BH-Adjusted *p* Value
Any MT positivity	52 (98.1)	9 (90.0)	0.294	0.490
MT-positive hepatocyte percentage			0.056	0.140
Negative	1 (1.9)	1 (10.0)		
<25%	6 (11.3)	4 (40.0)		
25–50%	5 (9.4)	0 (0.0)		
>50%	41 (77.4)	5 (50.0)		
MT staining intensity			0.013	0.065
Negative	1 (1.9)	1 (10.0)		
Mild	4 (7.5)	4 (40.0)		
Moderate	12 (22.6)	2 (20.0)		
Strong	36 (67.9)	3 (30.0)		
MT staining pattern			0.483	0.509
MT staining distribution			0.509	0.509

## Data Availability

The data presented in this study are available from the corresponding author upon reasonable request. The data are not publicly available due to privacy and ethical restrictions related to patient confidentiality.

## Abbreviations

The following abbreviations are used in this manuscript:

WD	Wilson disease
IHC	immunohistochemistry
AIH	autoimmune hepatitis
PSC	primary sclerosing cholangitis
MDR3	multidrug resistance protein 3
NAFLD	non-alcoholic fatty liver disease
MASLD	metabolic dysfunction-associated steatotic liver disease
MT	metallothionein
CAP	copper-associated protein
FFPE	formalin-fixed paraffin-embedded
EM	electron microscopy
PPV	positive predictive value
NPV	negative predictive value
BH	Benjamini–Hochberg
